# Study on the effect of phenoxyethanol–citric acid pretreatment for the enzymatic hydrolysis of bamboo residues

**DOI:** 10.3389/fbioe.2024.1483025

**Published:** 2024-10-03

**Authors:** Yan Cheng, Xiaoxue Zhao, Ruolin Li, Jili Liao, Caoxing Huang

**Affiliations:** Co-Innovation Center for Efficient Processing and Utilization of Forest Resources, College of Chemical Engineering, Nanjing Forestry University, Nanjing, China

**Keywords:** bamboo residues, phenoxyethanol, citric acid, enzymatic hydrolysis, biorefinery

## Abstract

This study investigated the biphasic phenoxyethanol–citric acid (PECA) pretreatment for bamboo residues (BRs) and its corresponding effects on the enzymatic hydrolysis performance. It is found that increasing the concentration of citric acid in the pretreatment system from 2.5% to 15% greatly enhanced the delignification and xylan removal for BRs. Consequently, the enzymatic hydrolysis yield of pretreated BRs significantly enhanced, increasing from 12.4% to 58.2% and 28.0%72.4% when the concentration of citric acid was increased from 2.5% to 15.0% at 160°C and 170°C, respectively. The characterization results from cellulose crystallinity, accessibility, and hydrophobicity of pretreated bamboo residues indicated that their changes possessed a beneficial performance on the enzymatic hydrolysis yield, which could result from the synergistic removal of lignin and xylan. The Chrastil model analysis showed that pretreatment at higher conditions resulted in the pretreated BRs possessing weaker diffusion resistance for cellulase, which is attributed to its higher enzymatic hydrolysis yield.

## 1 Introduction

The petrochemical industry caused the deterioration of the ecological environment, and such deterioration is becoming increasingly prominent (Domingo et al., 2020). In the face of the dual crisis of resources and the environment, it is urgent to replace traditional fossil fuel energy with more sustainable alternative fuels (Kumar Awasthi et al., 2022; Xu H. et al., 2023). Lignocellulosic biomass has significant potential to replace fossil fuels due to its abundant resource types, wide distributions, and renewability (Ijaz Ahmad et al., 2022; Qiu et al., 2024). Utilization of lignocellulose to produce bioenergy products, biochemicals, and biomaterials is one critical strategy for lowering the dependency on traditional fossil fuels (Haq et al., 2021; Singh et al., 2022; Xu et al., 2024). Bamboo is a kind of lignocellulosic biomass that is extensively found in Asian countries, particularly in China. China’s bamboo industry ranks high globally in terms of output, variety, and storage. At the same time, a significant amount of bamboo residues (BRs) is produced annually (Liu et al., 2012). These bamboo residues are currently underutilized or discarded by incineration and natural degradation. In contrast to treating bamboo residues as wastes, the bamboo residues containing a significant amount of carbohydrates [cellulose (∼39.9%) and hemicellulose (∼20.5%)] can provide potential opportunities as feedstocks for biofuel and bio-based material products (Wang et al., 2023). During the biorefinery process for bio-fuel production, enzymatic hydrolysis has been used as a common technology to degrade the polysaccharides into monosugars (glucose and xylose) that can be further converted to downstream bioenergy products (Hao et al., 2022; Liu et al., 2024).

Generally, the particle size, polymerization and crystallinity of cellulose, accessible surface area, and lignification degree are the factors that can affect the breakdown of biomass by enzymes (Xu C. Z. et al., 2023; Shakeel et al., 2024a). In addition, lignin in the lignocellulose cell wall can hinder the enzymatic hydrolysis by steric hindrance and non-productive cellulose adsorption for the enzyme to access the cellulose (Li et al., 2023; Huang et al., 2021). Therefore, all these factors need a better pretreatment technology to overcome, improving the fractionation rate of each component (cellulose, hemicellulose, and lignin) and aiming to achieve the excellent enzymatic hydrolysis yield of lignocellulosic biomass. Currently, various pretreatment technologies have already been applied for different lignocellulosic biomasses to enhance their enzymatic digestibility. However, pretreatment methods involving solvents like hydrochloric acid, sulfuric acid, and sodium carbonate have drawbacks during application, such as challenges in material recovery, environmental pollution, and severe corrosion (Qiao et al., 2021; Dharmaraja et al., 2023; Shakeel et al., 2024b). In this situation, the biphasic pretreatment system emerges as a green and novel technology for biomass to improve its enzymatic hydrolysis digestibility by removing lignin and hemicellulose in the cell wall. In addition, biphasic pretreatment could simultaneously retain most of the original cellulose in the pretreated substrate, making it the more ecologically friendly option than conventional pretreatment.

According to the Hansen solubility parameter theory based on the intermolecular forces (dispersion, polarity, and hydrogen bonding), a RED value between organic solvents and lignin of less than 1 means that the solvent can dissolve lignin well. It is reported that the RED value between phenoxyethanol and lignin is 0.9371, indicating that phenoxyethanol is an excellent lignin-soluble solvent (Zhang et al., 2019). Recently, coupling phenoxyethanol with an acid or alkaline solution as the biphasic pretreatment system is one of the more innovative pretreat technologies for biorefineries. For example, Zheng et al. (2021) found that by increasing the phenoxyethanol content in the phenoxyethanol–acid pretreatment system from 0:1 to 4:1, the lignin removal rate of bamboo residues could be increased from 29.4% to 91.6% and the hydrophobicity of bamboo residues could be reduced for the efficiency of enzymatic digestibility. Zhang et al. (2020) also pretreated rice straw with phenoxyethanol coupled with sodium hydroxide (NaOH), which achieved a lignin removal rate of 82.2% at 80°C. This pretreatment system reduced water consumption and energy consumption, improving the recovery and utilization rate of phenoxyethanol and washing wastewater (Zhang et al., 2020). The lignin removal rate (98.1%) and cellulose digestibility (74.5%) of bagasse were improved after mild organic solvent pretreatment with phenoxyethanol, mainly because the β-O-4 bond of lignin was degraded for increasing the total phenol content OH and H units (Li et al., 2023; Leong et al., 2021). Hence, it can be known that the phenoxyethanol in pretreatment technology could significantly remove and degrade the lignin in biomass. At present, phenoxyethanol is mostly coupled with inorganic acid, which cannot excellently remove lignin and hemicellulose. Therefore, phenoxyethanol coupled with organic acid can also be proposed for a biphasic pretreatment system. Typically, the phenoxyethanol–citric acid (PECA) pretreatment system has low cost, is non-toxic, and easily recovers the byproducts. For example, citric acid, which is phenoxyethanol-immiscible, can achieve static stratification, which is conducive to separate the phenoxyethanol and water-soluble degradation products in the pretreatment solution (Kim et al., 2016). However, few studies have coupled citric acid with phenoxyethanol as the biphasic pretreatment system for biomass to improve its enzymatic digestibility. In addition, immunolabeling and microscopy techniques were used to visually explore the migration patterns of hemicellulose and lignin within the cell walls of pretreated bamboo residues. This approach will also examine the dynamic adsorption changes between lignin and cellulase at the molecular level, providing both theoretical and practical foundations for the system.

In this work, phenoxyethanol coupled with citric acid was used as the pretreatment system to explore its performance and effects on the enzymatic digestibility of BRs. The various temperatures (160°C and 170°C) and citric acid concentrations (2.5%–15.0%) of pretreatment were investigated. To understand the mechanism of biphasic pretreatment for improving the enzymatic digestibility of pretreated BRs, the degree of delignification, xylan removal yield, cellulose accessibility, substrate hydrophobicity, crystallinity index, and crystal size were analyzed and correlated with the enzymatic hydrolysis yield. In addition, Chrastil’s model was used to study the diffusion-limited kinetics of cellulase with pretreated BRs, focusing on revealing the enzymatic hydrolysis mechanism of pretreated BRs (Chrastil, 1988). It is anticipated that this effort will present the knowledge of how PECA pretreatment can significantly improve the enzymatic hydrolysis of BRs.

## 2 Materials and methods

### 2.1 Materials

The bamboo (*Phyllostachys edulis*) residues were composed of 39.9% glucan, 20.5% xylan, 27.8% lignin (dry basis), and 11.8% extractive content. The moisture content of the samples was 13.4% (dry basis). Phenoxyethanol was purchased from Macklin (Shanghai, China), and citric acid was purchased from Shanghai Jiuyi Chemical Reagent Co., LTD. The bamboo residues were ground to 20–60 mesh sizes (0.28-mm–0.85-mm aperture) before pretreatment. Cellulase (CTec2) with 235 FPU/mL of filter paper activity was provided by Novozymes.

### 2.2 Phenoxyethanol–citric acid pretreatment for BRs

Phenoxyethanol (≥99.0%, w/w) and citric acid (≥99.5%, w/w) were mixed with a volume ratio of 3:1 to prepare a PECA pretreatment solution. The citric acid concentrations in the PECA solution were set as 2.5%, 5.0%, 7.5%, 10.0%, 12.5%, and 15.0%. For pretreatment, the BR (5 g) was processed with PECA (100 mL) at 160°C and 170°C for 60 min with a 1:20 solid-to-liquid ratio. After pretreatment, the liquid and solid components were separated using a funnel filled with G3 glassy sand. To remove residual chemicals in the pretreated solid, the pretreated BR was washed with water (approximately 60°C) to reach around pH 7. Then, the washed samples were stored for additional testing at 4°C. Regarding the pretreated liquids, heavy phases (citric acid solution) and light liquid phases (phenoxyethanol solution) of the PECA system were naturally separated after 24 h of standing. The dissolved xylo-oligosaccharides (XOS) and xylose in the citric acid solution were also analyzed to evaluate the pretreatment performance of the PECA system for BRs.

### 2.3 Enzymatic hydrolysis of BRs after pretreatment

The 20 FPU/g glucan cellulase, 2% (w/v) substrate, and 0.05 M citrate buffer with pH 4.8 was used for the enzymatic hydrolysis of the pretreated BR for 72 h at 50°C and 150 rpm. The glucose content of the samples (0.5 mL) obtained during the whole enzymatic hydrolysis process at various time intervals was measured to evaluate the enzymatic hydrolysis yield. The calculation Equation 1 is as follows:

$$Enzymatic\;hydrolysis\;yield\;\left(\%\right)=\frac{0.9\times Glucose\;in\;enzymatic\;hydrolysate\;\left(g\right)}{Glucan\;in\;pretreated\;BR\;\left(g\right)}\times 100\%.$$ (1)

### 2.4 Physicochemical properties of the pretreated BR

The Congo Red staining (DR28 staining) method was adopted to assess the accessibility of the pretreated BR (Inglesby and Zeronian, 2002). The pretreated BR (1%, w/v) was combined with dyes ranging from 0 to 4 g/L and then agitated at 50°C and 150 rpm for 24 h until the dye adhered to the substrate. The amount of dye adsorbed on the substrate was measured by using the Langmuir adsorption isotherm to assess the accessibility of the pretreated BR to cellulase. An X-ray diffractometer (XRD, Ultima IV, Rigaku, Inc.) with a scanning range of 5–50°/min and a scanning speed of 5°/min was used to assess cellulose crystallinity in the processed BR. The crystal size (B_hkl_) and crystallinity index (CrI) of cellulose were estimated based on published data and fitted by using the Mau Rietveld program (version 2.7) (Ling et al., 2017).

Rose bengal reagent (a hydrophobic dye) experiments were used to determine how hydrophobic the pretreated BR was. The pretreated BR of different loads (0.04, 0.08, 0.12, 0.16, and 0.20 g/L) was added to 20 mL of the citric acid buffer solution (50 mM, pH 4.8) containing rose red solution (40 mg/L). The mixture was then shaken for 2 h at 50°C and 150 rpm to facilitate reagent adsorption onto the substrate. The residual amount of the unabsorbed reagent in the supernatant was measured using a UV-vis spectrometer at 543 nm. The ratio of the baseline adsorption reagent residues to the quantity of free reagent remaining within the supernatant was calculated and adjusted to the concentration of the pretreated BR in the system. Eventually, the adjustment line inclination was calculated, explained as the pretreated BR surface hydrophobicity of (L/g).

### 2.5 Composition analysis

The compositions of raw and pretreated BRs were analyzed by using the US National Renewable Energy Laboratory (NREL) standard method (Sluiter et al., 2008). High-performance liquid chromatography (HPLC) was used to measure the sugar in the enzymatic hydrolysis liquid and acid hydrolysate from the composition analysis. The eluent was the 0.05 M sulfuric acid solution flowing at a rate of 0.6 mL/min at 50°C. Each experiment including pretreatment and enzymatic hydrolysis was carried out twice.

## 3 Results and discussion

### 3.1 Effects of the PECA pretreatment on changes in the components of the BR

Phenoxyethanol with different citric acid concentrations (2.5%–15%) and different temperatures (160°C and 170°C) was investigated to determine the optimal conditions for the PECA pretreatment of the BR. Table 1 shows that the unpretreated BR is composed of 39.9% cellulose, 20.5% xylan, and 27.8% lignin. When 2.5% citric acid was used in the phenoxyethanol–acid pretreatment system, the lignin and xylan removal rate for the BR at 160°C was 38.0% and 55.4%, respectively. This removal rate increased to 79.9% for xylan and 70.1% for lignin when the citric acid concentration was increased to 15% at 160°C. Zheng et al. (2021) also found that the proportion of acid concentration plays a crucial role in the biphasic phenoxyethanol pretreatment. At 170°C, the degree of delignification and removal yield of xylan increased to 93.3% and 86.0%, respectively. It is evident that in this biphasic pretreatment system, increasing the temperature and citric acid concentration greatly enhances the degree of delignification and xylan removal. It is notable that during the PECA pretreatment, increases in temperature and citric acid concentration had a minimal impact on the breakdown of cellulose, which can be identified by the majority of the retained glucan (76.4%–88.2%) in the pretreated BR.

**TABLE 1:** Changes in the compositions of BR after pretreatment.

Temp. (°C)	CA Concentration^a^ (%)	Composition (%)			Recovery yield (%)		Removal rate (%)	
		Glucan	Xylan	Lignin	Solid	Glucan	Xylan	Lignin
Unpretreated		39.9	20.5	27.8	—	—	—	—
160	2.5	42.5 ± 0.5	12.0 ± 0.1	22.6 ± 0.3	76.5 ± 0.5	81.4 ± 0.5	55.4 ± 0.2	38.0 ± 1.3
	5.0	47.2 ± 0.2	9.5 ± 1.6	20.8 ± 0.3	72.4 ± 0.3	85.6 ± 0.6	66.6 ± 5.5	45.8 ± 0.5
	7.5	55.3 ± 1.6	7.8 ± 0.2	15.1 ± 0.7	61.1 ± 0.5	84.7 ± 3.1	76.6 ± 0.8	66.9 ± 1.3
	10.0	51.3 ± 0.1	7.0 ± 0.1	13.3 ± 0.1	60.9 ± 0.6	78.1 ± 0.6	79.2 ± 0.8	71.0 ± 0.1
	12.5	57.2 ± 1.1	7.0 ± 0.2	16.7 ± 0.7	61.6 ± 0.4	88.2 ± 1.2	78.9 ± 0.3	63.1 ± 1.7
	15.0	53.6 ± 0.1	7.0 ± 0.1	14.2 ± 0.1	58.7 ± 0.4	78.8 ± 0.7	79.9 ± 0.2	70.1 ± 0.4
170	2.5	55.2 ± 0.1	8.0 ± 0.0	16.2 ± 0.3	60.7 ± 0.5	83.9 ± 0.7	76.3 ± 0.2	64.7 ± 1.0
	5.0	60.2 ± 0.4	6.6 ± 0.0	8.9 ± 0.7	57.4 ± 0.1	86.5 ± 0.7	81.4 ± 0.0	81.7 ± 1.3
	7.5	64.8 ± 0.0	5.2 ± 0.0	10.6 ± 1.3	54.1 ± 0.4	87.7 ± 0.6	86.2 ± 0.1	79.5 ± 2.5
	10.0	65.6 ± 0.5	5.4 ± 0.1	8.1 ± 0.1	52.6 ± 1.0	86.5 ± 2.3	86.1 ± 0.4	84.7 ± 0.4
	12.5	63.7 ± 0.1	5.6 ± 0.0	9.0 ± 0.2	53.7 ± 0.2	85.5 ± 0.1	85.2 ± 0.1	82.7 ± 0.3
	15.0	62.3 ± 0.2	2.8 ± 0.4	8.0 ± 1.8	48.9 ± 0.1	76.4 ± 0.1	93.3 ± 0.9	86.0 ± 3.2

^a^ Citric acid concentration (%) in the pretreatment system.

It has been reported that the pretreatment performance of a multi-component phenoxyethanol–acid pretreatment system is significantly superior to that of a single-phase pretreatment system, in the aspect of removing xylan (Chen et al., 2021; Zhang et al., 2020). Consequently, the yield of XOS in the pretreatment solution was also measured to evaluate the pretreatment performance. As shown in Table 2, with an increase in the content of citric acid in the phenoxyethanol–acid pretreatment system, the XOS yield showed a pattern of initially increasing and subsequently decreasing. For example, the XOS yield increased from 11.5% (2.5% acid concentration) to 24.7% (10% acid concentration) and then decreased to 16.0% (12.5% acid concentration) at 160 °C. Similarly, at 170°C, the XOS yield increased from 18.3% (2.5% acid concentration) to 21.7% (7.5% acid concentration) and then decreased to 9.0% (15% acid concentration). For XOS with various degrees of polymerization (DPs), the primary active ingredients are xylobiose (X2), xylotriose (X3), and xylotetraose (X4) (Huang et al., 2021). In this work, it can be observed that the obtained XOS was rich in X2–X4 fraction, indicating that the biphasic pretreatment system effectively converts xylan into XOS with a low DP, making it suitable for application in functional foods (Lan et al., 2021; Wen et al., 2021; Guo et al., 2019).

**TABLE 2:** XOS analysis of the pretreatment liquid at different temperatures and citric acid concentrations.

Temperature (°C)	CA Concentration^a^ (%)	X2–X6/g·L^−1^					X2–X6 (g·L^−1^)	XOS yield (%)
		X2	X3	X4	X5	X6		
160	2.5	0.914 ± 0.2	0.865 ± 0.1	0.769 ± 0.1	1.203 ± 0.1	0.961 ± 0.1	4.712 ± 0.1	11.492 ± 0.1
	5.0	0.376 ± 0.3	0.336 ± 0.1	0.321 ± 0.3	0.390 ± 0.1	0.231 ± 0.1	1.655 ± 0.2	4.036 ± 0.2
	7.5	2.223 ± 0.1	1.598 ± 0.0	1.045 ± 0.1	0.753 ± 0.1	0.548 ± 0.1	6.167 ± 0.1	15.041 ± 0.1
	10.0	3.026 ± 0.1	2.827 ± 0.1	1.798 ± 0.1	1.672 ± 0.3	0.799 ± 0.1	10.122 ± 0.1	24.688 ± 0.1
	12.5	2.156 ± 0.2	1.767 ± 0.2	1.109 ± 0.1	0.950 ± 0.1	0.586 ± 0.5	6.568 ± 0.3	16.020 ± 0.3
	15.0	2.668 ± 0.1	2.927 ± 0.1	1.721 ± 0.4	1.242 ± 0.1	1.053 ± 0.1	9.611 ± 0.2	23.441 ± 0.2
170	2.5	1.356 ± 0.1	1.693 ± 0.6	1.404 ± 0.1	1.684 ± 0.1	1.382 ± 0.1	7.519 ± 0.3	18.339 ± 0.3
	5.0	1.570 ± 0.1	2.527 ± 0.1	1.074 ± 0.0	0.800 ± 0.1	0.619 ± 0.1	6.590 ± 0.1	16.073 ± 0.1
	7.5	2.953 ± 0.0	2.774 ± 0.1	1.484 ± 0.1	0.988 ± 0.5	0.715 ± 0.0	8.914 ± 0.2	21.741 ± 0.1
	10.0	2.008 ± 0.0	1.497 ± 0.1	0.838 ± 0.1	0.537 ± 0.1	0.387 ± 0.1	5.267 ± 0.1	12.846 ± 0.6
	12.5	2.147 ± 0.1	1.325 ± 0.1	0.775 ± 0.1	0.614 ± 0.1	0.358 ± 0.1	5.219 ± 0.1	13.729 ± 0.1
	15.0	1.928 ± 0.2	0.976 ± 0.1	0.500 ± 0.8	0.291 ± 0.6	0.000 ± 0.1	3.695 ± 0.8	9.012 ± 0.4

^a^ Citric acid concentration (%) in the pretreatment system.

### 3.2 Impact of the PECA pretreatment on the enzymatic digestibility of the BR

**FIGURE 1:**
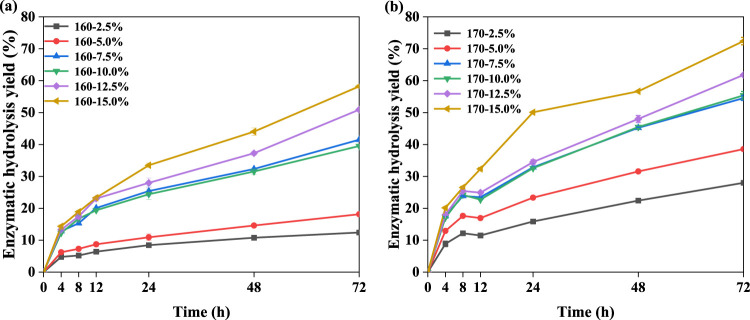
Enzymatic hydrolysis yield of pretreated BR at 160°C **(A)** and 170°C **(B)**.

**FIGURE 2:**
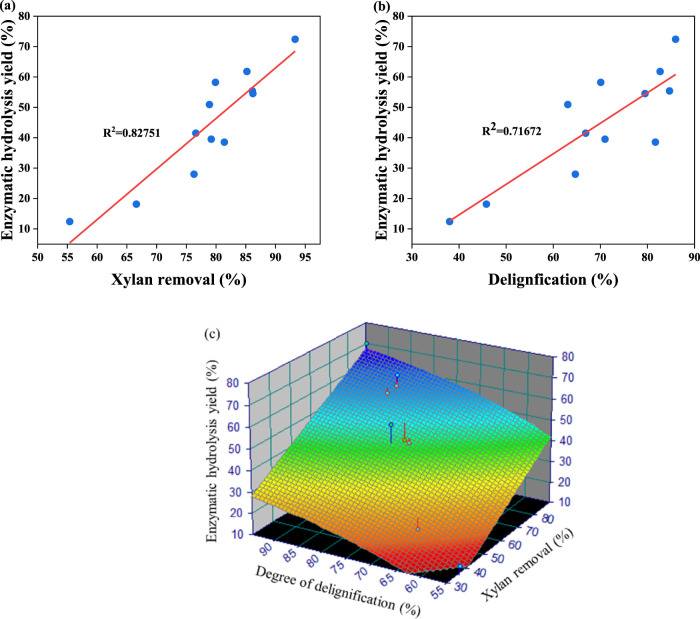
Correlations between the xylan removal yield **(A)** and degree of delignification **(B)** and enzymatic digestibility of pretreated BR. Regression 3D model used to predict the relationship between the enzymatic hydrolysis of pretreated BR and lignin/xylan removal **(C)**.

It has been identified that the increased amount of phenoxyethanol in the pretreatment system can make a contribution to enhance the removal performance for lignin, which can improve the glucose yields of pretreated biomass after enzymatic hydrolysis (Lai et al., 2020; Liu et al., 2024). Hence, the BR pretreated with the PECA system was subsequently treated by enzymatic hydrolysis by cellulase with 20 FPU/g glucan. Figures 1A, B show that the enzymatic hydrolysis yield of the pretreated BR could be enhanced from 12.4% to 58.2% (160°C) and from 28.0% to 72.4% (170°C) when the content of citric acid in the phenoxyethanol–citric acid pretreatment system increased from 2.5% to 15.0%. These findings indicated that PECA pretreatment could significantly increase the enzymatic hydrolysis efficiency of the BR. This shows that with greater delignification, the enzymatic hydrolysis of the pretreated BR significantly improved. Li et al. (2023) similarly concluded that the ketone/phenoxyethanol/water pretreatment system significantly enhanced the lignin removal rate (98.1%) and cellulase hydrolysis yield (74.5%) of sugarcane bagasse. Additionally, Zhu et al. (2023) proposed a synergistic effect between maleic acid and phenoxyethanol pretreatment, leading to the enzymatic hydrolysis yield of vinegar residue to 80.9%. Thus, the pretreatment of phenoxyethanol catalyzed by citric acid can significantly improve the degree of delignification and enzymatic hydrolysis performance of the BR (Dai et al., 2020). As shown in Figures 2A, B, delignification and xylan removal have a favorable correlation with the efficiency of enzymatic hydrolysis. It is well known that the exposed surface area of cellulose could increase the removing performance for lignin and xylan, which inevitably enhances its enzymatic digestibility (Zhu et al., 2023; Ma et al., 2018).

The cellulase hydrolysis yield serves as a critical indicator for evaluating pretreatment processes. The preceding data show that the degree of delignification is the major factor for the enzymatic digestibility of the pretreated BR with PECA, while hemicellulose removal is a secondary factor. Therefore, using TableCurve 3D software for the regression analysis of experimental data can predict the main factors for evaluating the enzymatic hydrolysis and pretreatment efficiency (Ouyang et al., 2021). The regression Equation 2 is as follows:

$$z=43.635555+0.65920625x-\frac{3359.8669}{y},$$ (2)

where *x* is the delignification degree, *y* is the xylan removal rate, and *z* is the enzymatic hydrolysis yield. The coefficient of the regression equation was 0.90. It could be concluded that the xylan removal rate and delignification have a high correlation with enzymatic hydrolysis efficiency. Otherwise, both x and y *p*-values were less than 0.001, demonstrating that these variables had a noteworthy impact on enzymatic hydrolysis. This finding aligns with the aforementioned enzymatic hydrolysis data, suggesting that the model can precisely forecast the enzymatic digestibility performance of the pretreated BR. This capability proves helpful in evaluating other conditions of the pretreatment process. Generally, removing lignin and xylan can enhance the cellulase-available surface area during enzymatic hydrolysis (Shen et al., 2019). The existing literature indicates that the extra level of delignification helps minimize non-specific binding between the remaining lignin and cellulase during the enzymatic hydrolysis process (Haq et al., 2020). Moreover, the remaining lignin and xylan in the pretreated substrate also affect the accessibility, cellulose crystallinity, and hydrophobicity of the pretreated substrate. These factors play a significant role in improving its enzymatic digestibility (Sheng et al., 2020). Consequently, it is essential to analyze the above characteristics of pretreated BR in detail to further explain the mechanism of PECA pretreatment.

In order to evaluate the recyclability of the biphasic pretreatment system of phenoxyethanol and citric acid, the phenoxyethanol and citric acid solutions were recovered (160°C and 170°C, 15%) and repeated for the pretreatment of bamboo residues four times. As shown in Table 3, the pretreated bamboo residues still contain cellulose-rich substrates, and the glucan content is 56.2%–66.2% (160°C) and 55.9%–64.6% (170°C), indicating that the recovered phenoxyethanol citric acid solution still shows a good pretreatment effect after four cycles of use. The enzymatic hydrolysis yield of bamboo residues pretreated with the recovered phenoxyethanol–citric acid solution decreased from 72.41% to 30.31% (170°C). This may be due to the fact that the recovered phenoxyethanol–citric acid solution may lose part of its activity due to degradation or pollution, thus affecting the enzymatic hydrolysis yield.

**TABLE 3:** Glucan content and enzymatic hydrolysis yields of phenoxyethanol-pretreated BR after different times of recycling.

Sample (%)	Times	Glucan (%)	Enzymatic hydrolysis yield (%)
160°C-15	1	60.7 ± 0.20	58.23 ± 0.55
	2	69.4 ± 0.80	35.42 ± 0.59
	3	56.2 ± 0.20	47.33 ± 0.56
	4	66.2 ± 1.00	32.28 ± 0.37
170°C-15	1	63.2 ± 0.20	57.38 ± 0.04
	2	64.6 ± 0.00	72.41 ± 1.14
	3	64.0 ± 0.00	45.08 ± 0.28
	4	55.9 ± 0.60	44.44 ± 0.45

“Times” refers to the number of times phenoxyethanol is recovered.

### 3.3 Structural characterization of pretreated BR and its effects on the enzymatic hydrolysis yield in pretreated BR

Measuring the crystalline characteristics of cellulose in the BR can show how the PECA pretreatment affects the enzymatic hydrolysis yield. Table 4 shows that the crystallinity of the BR increased from 52.5% to 68.0% at 160°C and from 59.5% to 69.9% at 170°C when the citric acid content was increased from 2.5% to 15.0% in the pretreatment system. Generally speaking, the higher the crystallinity of cellulose (CrI), the less the site of action between cellulose and enzymes, the lower the enzymatic hydrolysis yield. In this study, with the increase in the citric acid concentration in the pretreatment process, the crystallinity of cellulose was positively proportional to the enzymatic hydrolysis yield, and the trend increased. This phenomenon may be due to the removal of hemicelluloses and lignin in the treatment process, which increases the contact area between phenoxyethanol and cellulose, promotes the dissolution of phenoxyethanol to cellulose, effectively breaks the crystalline structure of cellulose, and realizes the efficient conversion of cellulose hydrolysis to glucose in the mild enzymatic hydrolysis process (Ling et al., 2017; Lin et al., 2021; Jiang et al., 2020). Generally, the crystal size of cellulose can be considered the factor to influence the enzymatic hydrolysis yield of biomass.

**TABLE 4:** Changes in the physicochemical properties of pretreated BR under different conditions.

Temperature (°C)	CA Concentration^a^ (%)	Hydrophobicity (L/g)	Accessibility (mg/g)	CrI (%)	B_hkl_ (nm)
160	2.5	2.60	364.12	52.55	2.57
	5.0	2.47	360.72	56.40	2.67
	7.5	1.88	392.92	56.19	2.37
	10.0	1.52	392.88	60.23	2.73
	12.5	1.75	405.37	62.66	2.84
	15.0	1.70	483.84	68.04	3.15
170	2.5	2.45	339.07	59.53	2.67
	5.0	1.56	378.04	63.41	2.93
	7.5	1.04	397.65	62.40	2.81
	10.0	0.32	401.23	64.30	2.98
	12.5	0.27	410.49	65.89	2.94
	15.0	0.69	492.75	69.88	3.19

^a^ Citric acid concentration (%) in the pretreatment system.

**FIGURE 3:**
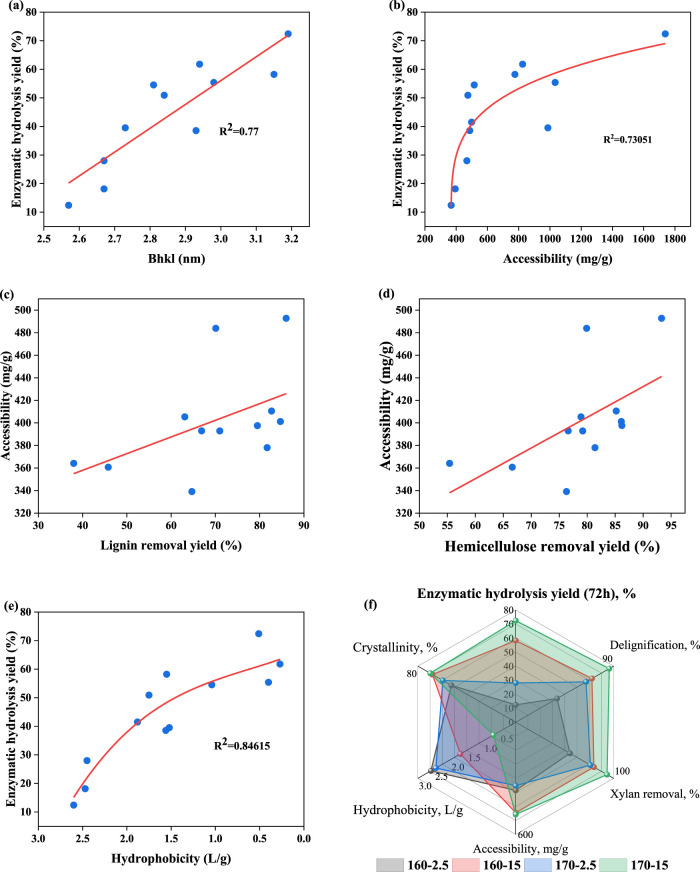
Correlations between the crystal size **(A)**, cellulose accessibility **(B)**, degree of delignification **(C)**, xylan removal yield **(D)**, substrate hydrophobicity **(E)**, and enzymatic hydrolysis yield. Radar images of delignification, xylan removal, accessibility, hydrophobicity, crystallinity, and glucose yield **(F)**.

Table 4 shows that the crystal size of pretreated BR (B_hkl_) showed a regular trend of change with increasing citric acid concentration. Specifically, the B_hkl_ values of cellulose in pretreated BR increased from 2.57 nm to 3.15 nm (160°C) and from 2.67 nm to 3.19 nm (170°C) when increasing the citric acid concentration from 2.5% to 15%. This phenomenon may be due to the increased pretreatment efficiency, which results in the re-folding of cellulose chains, thereby increasing the crystallite size (Keshk, 2015; Guo et al., 2024). Considering the B_hkl_ value of cellulose and enzymatic digestibility, the crystal size of the sample at 160°C and 170 °C demonstrated a relatively good correlation with the enzymatic hydrolysis yield (R^2^ = 0.74) (Figure 3A), which is consistent with the data obtained by Lin et al. In other words, the change in the crystal size of bamboo cellulose after deep eutectic solvent (DES) pretreatment was 2.71–3.31 nm, and the R^2^ value of crystal size and enzymatic hydrolysis yield was 0.76, indicating a good correlation (Lin et al., 2020). Overall, this is in line with the previous literature (Ling et al., 2020; Wang et al., 2022), where the enhanced enzymatic digestibility of cellulose after deep eutectic solvent pretreatment was due to the promoted microfiber morphology destruction and crystal shortening. In summary, the biphasic pretreatment system can expand the microfibers in cellulose and increase the cellulose surface area during the expansion process, which significantly enhances enzymatic hydrolysis.

It has been demonstrated that lignin and xylan removal during pretreatment can improve the accessibility of enzymes to hydrolysis substrates (Zheng et al., 2023). As shown in Table 4 and Figure 3B, the accessibility of pretreated BR increased from 364.12 mg/g to 483.84 mg/g (160°C) and from 339.07 mg/g to 393.46 mg/g (170°C) when the citric acid content was increased from 2.5% to 15.0% in the pretreatment system. This trend is consistent with the changes observed in lignin and xylan removal (Table 1). Delignification (Figure 3C) and xylan removal (Figure 3D) displayed a strong linear connection with accessibility. It is indicated that as the pretreatment became more intense (increased citric acid concentration) with more lignin removed from the BR surface, more cellulose was exposed, thus further improving cellulase’s accessibility to cellulose. As shown in Figure 3B, there is a gentle curve (an “L” curve in reverse) between cellulose accessibility and enzymatic hydrolysis yield, demonstrating that an increase in cellulose accessibility positively impacted enzymatic hydrolysis. However, as soon as the pretreatment temperature reached 170°C, the correlation between enzymatic hydrolysis yield and cellulose accessibility revealed a decreasing trend. The results showed that cellulose accessibility was no longer the limiting factor for enzymatic hydrolysis yield when reaction conditions reached a certain limit. This may be because pretreatment at lower temperatures resulted in significant delignification and xylan removal, making cellulose accessibility the main factor affecting enzymatic hydrolysis efficiency (Lai et al., 2020).

Table 4 shows that the hydrophobicity of pretreated BR decreased from 2.60 to 1.70 at 160°C and from 2.45 to 0.69 at 170°C when the citric acid concentration was increased from 2.5% to 15.0% in the pretreatment. This result is consistent with the research conducted by Zheng et al. (2021). Tang et al. (2020) also indicated that substrates with higher hydrophobicity can cause more ineffective cellulase binding and lower the effectiveness of enzymatic hydrolysis. As shown in Figure 3E, the hydrophobicity of pretreated BR was linearly correlated with its enzymatic hydrolysis yield (R^2^ = 0.77). This indicated that there was an important correlation between the decrease in hydrophobicity and increase in the enzymatic digestibility of pretreated BR. To further analyze the relationship between pretreated substrate characteristics and enzymatic hydrolysis, Figure 3F shows a comprehensive overview of enzymatic hydrolysis yield and the changed characteristics of BR caused by PECA pretreatment. Figure 3F shows that the enzymatic hydrolysis performance of pretreated BR may be influenced by multiple substrate characteristic factors. With the increase in pretreatment intensity, the substrate’s hydrophobicity had a more significant impact on the enzymatic digestibility of the pretreated BR. This result indicated that the hydrophobic relationship between lignin and cellulase is a primary factor affecting the enzymatic hydrolysis performance, thereby exerting a detrimental impact on the enzymatic hydrolysis process. Similar conclusions were also drawn by Nakagame et al. (2011). Overall, the biphasic phenoxyethanol–acid pretreatment system significantly disrupted the complex structure of BR by removing xylan and lignin, leading to an increased substrate–cellulose contact area and amorphous cellulose region breakdown, ultimately improving the enzymatic hydrolysis yield.

### 3.4 Diffusion restriction kinetics of cellulase by PECA-pretreated bamboo residues

**FIGURE 4:**
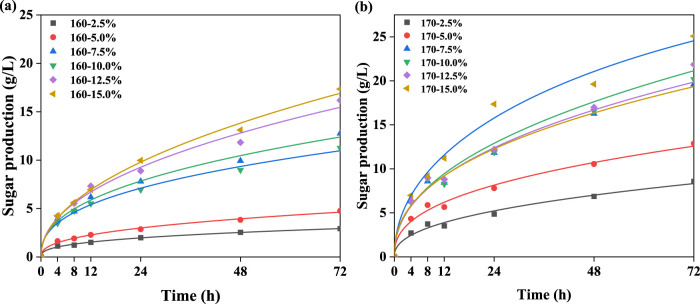
Fitting graphs of glucose concentration and time under different conditions at 160°C **(A)** and 170°C **(B)**.

During the enzymatic hydrolysis process, the kinetic behavior of cellulase diffusion to pretreated substrates can be considered to evaluate the efficiency of the pretreatment system. To further understand the effect of pretreated substrates on cellulase diffusion during enzymatic hydrolysis, the Chrastil model (Chrastil, 1988) was used to determine the kinetic parameters n and k, as shown in Table 5. In this model, n is the structural diffusion resistance constant, and k is the rate constant. Which are calculated based on the sugar production of the pretreatment (Figures 4A, B).

**TABLE 5:** Kinetic parameters of the Chrastil model obtained under different pretreated conditions.

Temperature (°C)	CA Concentration^a^ (%)	*k* (g·L^−1^h^−1^)	*n*	R^2^
160	2.5	8.39406 × 10^−4^	0.36	0.99999
	5.0	2.27544 × 10^−4^	0.40	0.99997
	7.5	2.35584 × 10^−4^	0.42	0.99979
	10.0	2.05186 × 10^−4^	0.40	0.99991
	12.5	2.31916 × 10^−4^	0.47	0.99934
	15.0	1.98094 × 10^−4^	0.50	0.99974
170	2.5	2.43888 × 10^−4^	0.42	0.99978
	5.0	2.44633 × 10^−4^	0.40	0.99968
	7.5	2.47852 × 10^−4^	0.41	0.99949
	10.0	2.74661 × 10^−4^	0.43	0.99922
	12.5	2.23495 × 10^−4^	0.45	0.99900
	15.0	3.41448 × 10^−2^	0.48	0.99845

^a^ The concentration of citric acid in the entire system.

According to Table 5, the results for all pretreatment systems (R^2^ > 0.99) showed that the enzymatic hydrolysis kinetics model of pretreatment BR was suitable and accurate to predict the enzymatic hydrolysis yield of pretreatment BR, which was helpful for evaluating phenoxyethanol–citric acid biphasic pretreatment. Lin et al. (2021) proposed that the resistance constant was associated with the spatial structure of the enzymatic hydrolysis system. The greater inhibitory effect of substrates on the cellulase hydrolysis system resulted in more pronounced diffusion resistance (lower n value). In this work, the n value increased from 0.36 to 0.50 at 160°C and from 0.42 to 0.48 at 170°C with the increasing citric acid concentration in the pretreatment system. This suggested that the enhanced diffusion resistance could be reduced to the higher pretreatment intensity, leading to a more pronounced delignification of the substrate, thereby promoting the process by which enzymes diffuse into the structure of the substrate. Generally, a lower k value indicated stronger resistance of cellulose to enzyme hydrolysis, leading to a reduction in the reaction between enzymes and substrates (Chrastil, 1988).

Table 5 shows that temperature was a key factor affecting the k-value of enzymatic hydrolysis. Specifically, at 160°C, there was no clear trend in the variation in k with increasing citric acid concentration. At 170°C, as the citric acid concentration increased, there was an overall increasing trend in k, but a decrease was observed at 12.5% acid concentration, indicating that increased pretreatment intensity could effectively weaken the resistance between cellulase and the substrate, thereby enhancing the cellulase hydrolysis of pretreated BR. Moreover, the difference in the k-value also suggested that cellulase hydrolysis was a rate-limiting reaction, and its diffusion behavior could be affected by various factors, possibly as a comprehensive result of multiple substrate characteristics (Nill et al., 2018). Overall, PECA pretreatment reduced the cellulase's resistance to substrates and weakened the diffusion resistance in the enzymatic hydrolysis system, facilitating the penetration of cellulase during subsequent hydrolysis.

### 3.5 Mass balance of bamboo residue pretreatment

**FIGURE 5:**
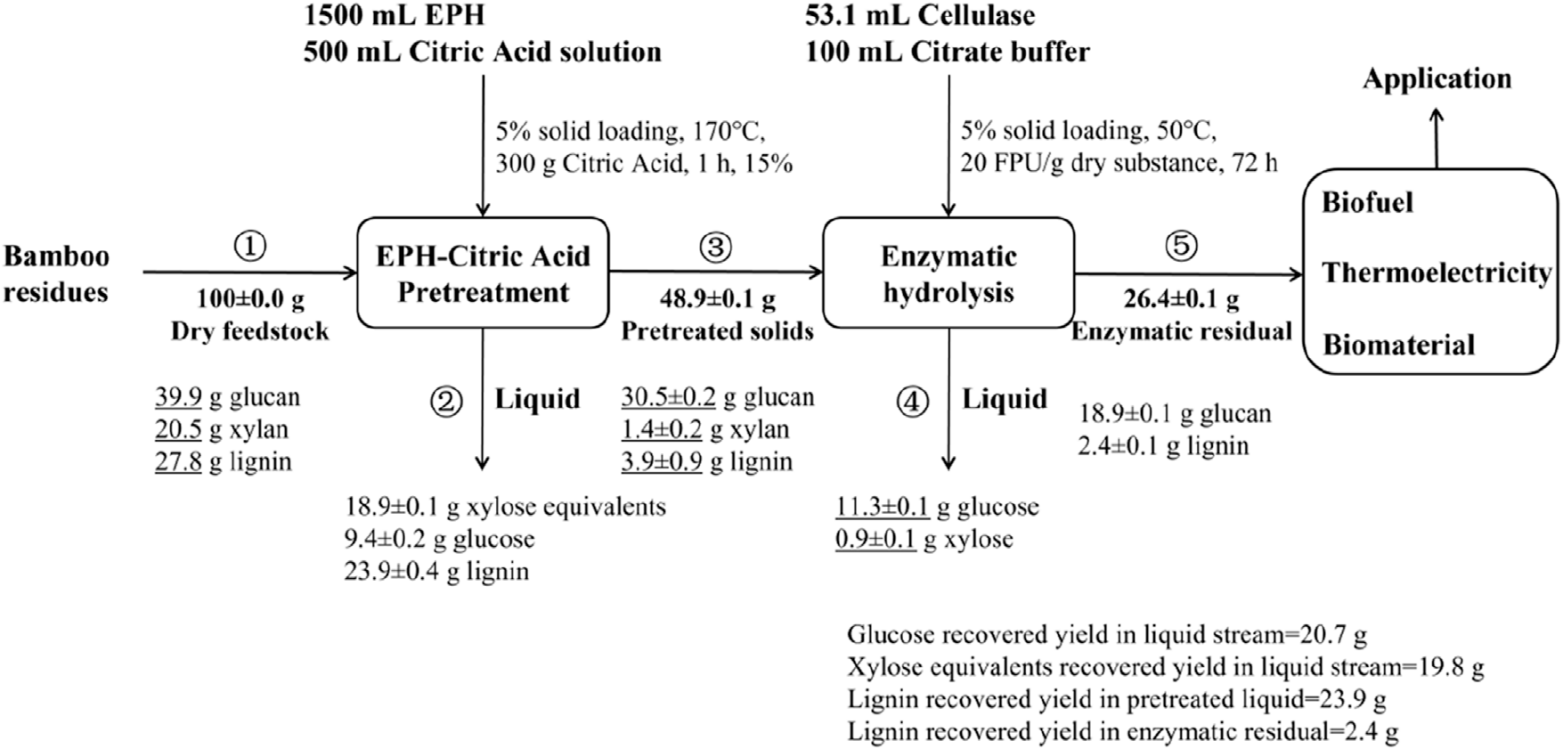
Quality balance of phenoxyethanol–citric acid pretreatment. Balance is adjusted to 100 g starting biomass.

In this work, the biphasic pretreatment system composed of phenoxyethanol and citric acid efficiently separated lignin and xylan from the BR. To verify the feasibility of the techniques, we investigated the process mass balances during the machining process (Figure 5). The mass balance of biphasic pretreatment was based on 100 g BR. After 1 h PECA treatment at 170°C, 100 g solids were finally obtained, with 30.5 g glucan, 1.4 g xylan, and 3.9 g lignin. This suggested that xylan and lignin have been eliminated significantly during the biphasic pretreatment. In addition, the mass balances of the solvents were established for this investigation (Cheng et al., 2023). After biphasic pretreatment, the aqueous phase of 2,000 mL of pretreated liquid (with negligible loss) contains approximately 18.9 ± 0.1 g xylose equivalent. Most of the hemicellulose was degraded into monosaccharides, which existed in the water phase of the pretreatment liquid. The theoretical glucose content obtained by enzymatic saccharification was 18.9 g. Overall, phenoxyethanol is almost immiscible with water and can still be used as a new solvent system after repeated recovery and has shown strong lignin and xylan removal ability in pretreatment. The feature of phenoxyethanol holds a great deal of importance for the efficient preparation of biomass sugar from BR.

## 4 Conclusion

A novel biphasic solvent pretreatment system utilizing phenoxyethanol and citric acid was introduced for BR. It can efficiently eliminate hemicellulose and lignin from BR and increase its enzymatic hydrolysis yield (72.41%). In addition, this pretreatment could increase cellulose crystallinity, crystal size, and accessibility in the BR while reducing the hydrophobicity of pretreated BR, thereby facilitating enzymatic hydrolysis, which could result from the synergistic removal of lignin and xylan from BR. Kinetic parameters obtained from the Chrastil model in this pretreatment could weaken the diffusion resistance between cellulase and the substrate, thereby enhancing the cellulase hydrolysis of pretreated BR.

## Data Availability

The raw data supporting the conclusion of this article will be made available by the authors, without undue reservation.
